# Sensitivity of liquid clouds to homogenous freezing parameterizations

**DOI:** 10.1002/2014GL062729

**Published:** 2015-03-13

**Authors:** Ross J Herbert, Benjamin J Murray, Steven J Dobbie, Thomas Koop

**Affiliations:** 1 School of Earth and Environment, University of LeedsLeeds, UK; 2 Faculty of Chemistry, Bielefeld UniversityBielefeld, Germany

**Keywords:** homogeneous nucleation, ice nucleation, cloud glaciation, droplet freezing, mixed-phase clouds, cloud ice

## Abstract

Water droplets in some clouds can supercool to temperatures where homogeneous ice nucleation becomes the dominant freezing mechanism. In many cloud resolving and mesoscale models, it is assumed that homogeneous ice nucleation in water droplets only occurs below some threshold temperature typically set at −40°C. However, laboratory measurements show that there is a finite rate of nucleation at warmer temperatures. In this study we use a parcel model with detailed microphysics to show that cloud properties can be sensitive to homogeneous ice nucleation as warm as −30°C. Thus, homogeneous ice nucleation may be more important for cloud development, precipitation rates, and key cloud radiative parameters than is often assumed. Furthermore, we show that cloud development is particularly sensitive to the temperature dependence of the nucleation rate. In order to better constrain the parameterization of homogeneous ice nucleation laboratory measurements are needed at both high (>−35°C) and low (<−38°C) temperatures.

**Key Points:**

- Homogeneous freezing may be significant as warm as −30°C
- Homogeneous freezing should not be represented by a threshold approximation
- There is a need for an improved parameterization of homogeneous ice nucleation

## 1. Introduction

Clouds play an integral role in the Earth's energy budget [*Boucher et al.*, 2013] and can be sensitive to the presence of ice; however, an explicit understanding of ice formation processes and appropriate representation in models is currently lacking. Cloud droplets are frequently observed to supercool to temperatures approaching −35°C and even below [*Choi et al.*, 2010; *de Boer et al.*, 2011; *Rosenfeld and Lensky*, 1998; *Rosenfeld and Woodley*, 2000; *Westbrook and Illingworth*, 2011]. At these extreme supercooled temperatures, it is known that freezing can occur via the homogeneous nucleation of ice [*Murray et al.*, 2010; *Riechers et al.*, 2013], and this process therefore needs to be appropriately represented in cloud models.

Laboratory measurements show that homogeneous ice nucleation rates are strongly temperature dependent, and therefore, it has been commonly assumed that homogeneous freezing in cloud simulations may be approximated by a step function at a threshold temperature. A number of microphysics schemes currently use this approach with the threshold set at either −38 or −40°C [e.g., *Forbes and Ahlgrimm*, 2014; *Kong and Yau*, 1997; *Lim and Hong*, 2010; *Morrison et al.*, 2005; *Reisner et al.*, 1998; *Thompson et al.*, 2008]. However, there is a finite but uncertain nucleation rate at temperatures warmer than these thresholds; hence, some schemes, albeit the minority, use parameterizations based on experimental measurements [e.g., *Cotton and Field*, 2002; *Lynn et al.*, 2005; *Milbrandt and Yau*, 2005; *Seifert and Beheng*, 2006; *Walko et al.*, 1995]; see Table S1 in the supporting information for a summary of microphysics schemes in the literature. Using a cloud resolving model, *Fan et al.* [2010] tested several existing heterogeneous and homogeneous freezing parameterizations including a relatively warm threshold of −36°C and found that deep convective anvil properties were sensitive to the different representations. In this paper we use a parcel model with detailed microphysics to show that supercooled liquid clouds formed at a range of updraft speeds are sensitive to the numerical representation of homogeneous freezing and that the commonly used threshold approximation of −40°C may be inappropriate.

## 2. Homogeneous Parameterizations and Model Description

### 2.1. Parameterizations

**Figure 1:**
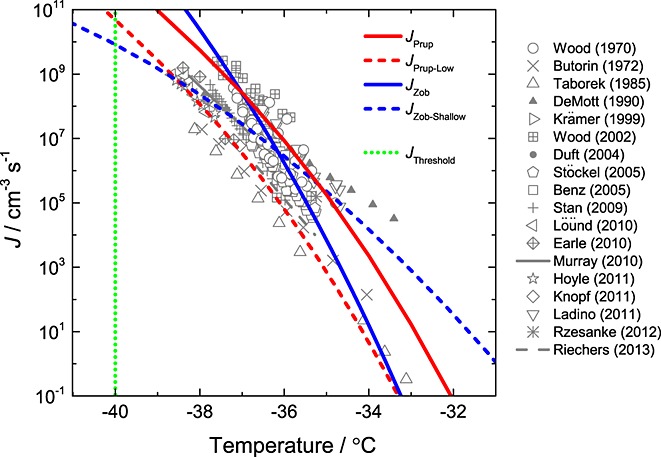
Homogeneous ice nucleation rate coefficient data determined from laboratory measurements (grey symbols and lines) and estimated using CNT following *Pruppacher* [1995] and *Zobrist et al*. [2007] (solid red and blue lines). Additional parameterizations adapted from the CNT-based lines and constrained by the measurements are shown as dashed red and blue lines. A threshold freezing approximation of −40°C is also included, shown as a temperature-independent step function (green dotted line). The data are taken from *Benz et al*. [2005], *Butorin and Skripov* [1972], *Demott* [1990], *Duft and Leisner* [2004], *Earle et al*. [2010], *Hoyle et al*. [2011], *Knopf and Rigg* [2011], *Krämer et al*. [1999], *Ladino et al*. [2011], *Lüönd et al*. [2010], *Murray et al*. [2010], *Riechers et al*. [2013], *Rzesanke et al*. [2012], *Stan et al*. [2009], *Stöckel et al*. [2005], *Taborek* [1985], *Wood and Walton* [1970], and *Wood et al.* [2002]. A table of equations for each parameterization can be found in Table S2 in the supporting information.

A summary of laboratory measurements of the homogeneous ice nucleation rate coefficient, *J* (nucleation events per unit volume and unit time for ice in pure water), as a function of temperature is shown in Figure 1. We neglect nucleation at droplet surfaces, since measurements indicate that it is not important for cloud-sized droplets [*Duft and Leisner*, 2004]. The data in Figure 1 show that *J* increases steeply over 5 orders of magnitude within ∼3°C, and there is a considerable spread in *J* values at all temperatures, along with a range in measured temperature (*T*) dependences (i.e., the gradient of *J* versus *T*).

In order to assess the sensitivity of homogeneous ice nucleation in clouds to the uncertainty in the measured rate coefficients, we have used a number of parameterizations which are consistent with the data (see Figure 1). *Pruppacher* [1995] and *Zobrist et al.* [2007] used classical nucleation theory (CNT) to estimate values of *J*. These parameterizations are shown as solid, colored lines in Figure 1 and are individually referred to as *J*_Prup_ and *J*_Zob_. A second set of parameterizations was developed here to represent sensitivity to the absolute value of *J* and the *T* dependence of *J*, referred to as *J*_Prup-Low_ and *J*_Zob-Shallow_, respectively. The two parameterizations, shown as dashed colored lines in Figure 1, are based on *J*_Prup_ and *J*_Zob_ and are constrained to experimental measurements. The parameterizations to describe the *J* measurements are collectively referred to as *J*_CNT_; the equations used for each parameterization are shown in Table S2 in the supporting information. Threshold freezing approximations have been used in previous modeling studies with temperatures of –35, –38, or −40°C (see Table S1 in the supporting information). For this study a value of −40°C was used in the threshold simulations due to its common use and is referred to as *J*_Threshold_; the dotted green line in Figure 1 represents this function.

### 2.2. Description of Model

The simulations were performed using the Met Office Kinematic Driver (KiD) model. The KiD model described by *Shipway and Hill* [2012] is a one- or two-dimensional dynamical framework within which the dynamics and optional microphysical forcings are prescribed throughout the simulation. For the purpose of this study, the one-dimensional version was used with prescribed conditions so that a constant cooling rate was achieved. Only a single grid point was considered, and hydrometeor sinks were limited to precipitation, resulting in an idealized adiabatic parcel model simulating a trajectory through the atmosphere. The simulations were initialized under saturated conditions (relative humidity of 100% with respect to liquid water) at a temperature of −5°C and the parcel lifted along a saturated adiabat; the parcel therefore contained a population of water droplets soon after the simulation started. An additional set of simulations were initialized at −30°C to simulate a shallower cloud.

*Wright and Petters* [2013], *Vali* [2014], and *Herbert et al.* [2014] have shown that the *T* dependence of the nucleation rate coefficient determines the stochastic time-dependent behavior of ice nucleation; therefore, the simulations were run under a range of cooling rates to additionally test the sensitivity of homogeneous freezing to the cooling rate. The Thompson two-moment bulk microphysics scheme was chosen from a number of existing embedded options in the KiD model; the scheme is one of several coupled to the Weather Research and Forecasting model and has been shown to be representative alongside other microphysics schemes within the KiD model framework [*Shipway and Hill*, 2012]. The scheme, described in full by *Thompson et al.* [2008], predicts cloud water, rain, cloud ice, graupel, and snow and includes a detailed treatment of in-cloud interactions between all hydrometeor species and water vapor. The size distributions of each hydrometeor species are represented by Marshall-Palmer distributions except for snow which is described using a combined exponential and gamma distribution. Cloud droplets, not described by the explicit activation of aerosols, are constrained to a concentration of 200 cm^−3^, representing a relatively clean cloud. In each simulation, the primary production of ice was limited to homogeneous freezing of cloud and rain droplets only.

In the *J*_Threshold_ simulations, all liquid water is converted into ice over a single time step once the threshold temperature is reached. For the *J*_CNT_ simulations (*J*_Prup_, *J*_Zob_, *J*_Prup-Low_, and *J*_Zob-Shallow_), the number of liquid droplets (both cloud and rain droplets) that freeze in each time step Δ*t* is a function of *T* and droplet size and is calculated following


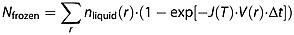
(1)

where *n*_liquid_(*r*) is the number of droplets of radius *r*, *V* is the droplet volume, and *J* is the homogeneous ice nucleation rate coefficient at *T*. For each prescribed cooling rate, the simulations were run consecutively with each *J*(*T*) parameterization. In each set of simulations, an equivalent updraft speed, *w*, was calculated assuming a wet adiabatic lapse rate of −5.5°C km^−1^, resulting in the range of 0.04 ≤ *w* ≤ 30 m s^−1^.

## 3. Results and Discussion

**Figure 2:**
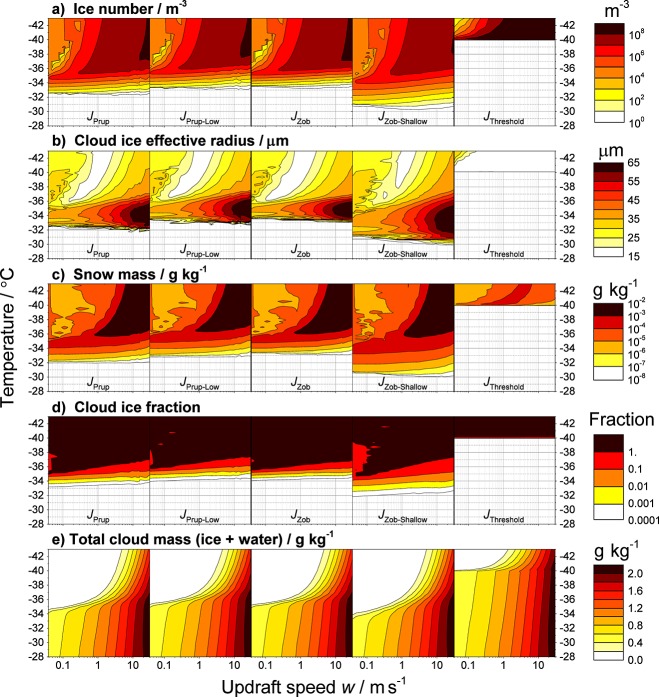
Simulated one-dimensional evolution of cloud variables as a function of constant updraft speed using different homogeneous freezing representations. Variables include (a) cloud ice particle number concentration, (b) cloud ice particle effective radius (expression for the cross-section area weighted mean radius), (c) snow mass mixing ratio, (d) cloud ice fraction (ratio of ice mass mixing ratio to total hydrometeor mass mixing ratio), and (e) total cloud mass mixing ratio (all ice and water species). The first four columns demonstrate sensitivity of the cloud to the laboratory-constrained *J*(*T*) parameterizations, and the final column shows simulations using a threshold approximation of −40°C at which point all liquid instantly freezes.

Figure 2 shows the one-dimensional evolution of the ice number concentration, cloud ice effective radius, snow mass mixing ratio, cloud ice fraction (defined as the ratio of ice mass to total cloud mass), and the total cloud mass mixing ratio as a function of parcel updraft speed. The first four columns in Figure 2 represent the *J*_CNT_ simulations using *J*_Prup_, *J*_Zob_, *J*_Prup-Low_, and *J*_Zob-Shallow_, and the final column is using *J*_Threshold_, where all liquid water freezes at −40°C. The cloud liquid water content (LWC) prior to freezing can be inferred from the total cloud mass shown in Figure 2e at *T* = −30°C. The simulated LWC ranges from ∼0.4 to 2.0 g kg^−1^ (∼0.5 to 3 g m^−3^) on increasing *w* from ∼0.1 to 20 m s^−1^. This range is in agreement with LWC measurements from convective clouds made by *Draginis* [1958] and highly supercooled clouds by *Rosenfeld and Woodley* [2000]. In the analysis of the simulations, we define the first ice as a concentration of ≥1 m^−3^; however, it is worth noting that this small concentration increases by several orders of magnitude within a single degree. In all simulations, both cloud droplets and rain droplets are present and contribute to the glaciation of the cloud via homogeneous freezing.

### 3.1. Suitability of Threshold Approximation

A clear distinction between the *J*_Threshold_ and *J*_CNT_ simulations is evident in the evolution of all cloud variables. The first ice occurs >6°C warmer in the *J*_CNT_ simulations with the most extreme case 10°C warmer than *J*_Threshold_, which corresponds to ∼1800 m difference in altitude. Due to the rapid freezing in the *J*_Threshold_ simulations, the considerable differences observed are due to the increased amount of time for secondary processes, such as ice sedimentation, hydrometeor interactions, and depositional growth, to occur in the *J*_CNT_ model runs. These combined processes affect both the size distribution of the ice particles and the total cloud mass mixing ratio, as seen in Figures 2b and 2e, suggesting a considerable impact on the cloud radiative properties of the evolving and glaciated cloud. The smaller ice particles may also increase the cloud lifetime, thus enhancing this impact. In these simulations, snow and graupel are minor constituents; however, they also demonstrate sensitivity to the representation of freezing, albeit indirectly, as shown in Figure 2c for snow. The simulations show a degree of cooling rate dependence, which is primarily due to the relationship between *w* and supersaturation, and the enhanced time-dependent processes at low *w*; this dependence is considerably enhanced in the *J*_CNT_ simulations as evident in Figure 2d. At low *w* (∼0.1 m s^−1^), the cloud fully glaciates between 3 and 5°C warmer than in the *J*_Threshold_ simulation depending on the *J*_CNT_ function used. A second set of simulations initialized at −30°C, thus simulating a shallower cloud, are included in Figure S1 in the supporting information. There is considerably less cloud LWC (<0.15 g kg^−1^) in these shallow clouds; however, the *J*_CNT_ simulations continue to produce ice up to 8°C warmer than using *J*_Threshold_, and similarly, the evolution of other cloud properties is impacted. The differences between *J*_Threshold_ and *J*_CNT_ simulations clearly show that the threshold approximation is unable to represent the freezing behavior observed in simulations using the parameterizations constrained by laboratory data.

The results presented in Figure 2 should change the way we view homogeneous freezing. It is generally assumed that homogeneous freezing is a negligible process at temperatures above ∼−38°C when compared to heterogeneous ice nucleation; however, these results show that there may be considerable competition between the two nucleation modes. Observations and experiment-based extrapolations report atmospheric ice nuclei concentrations on the order of 10^2^ to 10^5^ m^−3^ at −34°C [*DeMott et al.*, 2010; *Murray et al.*, 2012]. Inspection of Figure 2a suggests that homogeneous freezing may provide between 10 and 10^6^ m^−3^ ice particles at −34°C depending on the parameterization and updraft speed. Hence, homogeneous ice nucleation competes with heterogeneous ice nucleation at temperatures well above −38°C.

### 3.2. Sensitivity to Parameterization

Figure 2 demonstrates that the simulated cloud is sensitive to *J*_CNT_, in particular the *T* dependence of *J*. A systematic decrease in the absolute value of *J* from *J*_Prup_ to *J*_Prup-Low_ results in clouds which have approximately the same properties but are offset by ∼200 m in altitude, which demonstrates a relatively weak sensitivity. In contrast, a change in the *T* dependence from the steep *J*_Zob_ to shallow *J*_Zob-Shallow_ parameterization causes an onset of ice up to ∼3°C warmer. Unlike a change in the absolute value of *J*, a change in the *T* dependence does not have a simple linear effect on the cloud evolution. In the simulations, cloud evolution following ice onset proceeds more slowly in the *J*_Zob-Shallow_ simulations than *J*_Zob_ and allows more time for secondary processes and in-cloud interactions to occur before complete glaciation. The result is an increased ice depositional growth, leading to larger ice particles and increased cumulative sedimentation of ice. These changes may impact the radiative properties of the mixed phase and glaciated cloud as inferred from Figures 2b and 2e. As shown by *Herbert et al.* [2014], the *T* dependence of *J* controls the response of ice production to changes in cooling rate. This behavior can be seen in Figure 2d; the glaciation temperature (where cloud ice fraction = 1) is more dependent on *w* in the *J*_Zob-Shallow_ simulations than any other *J*_CNT_. The result is a longer-lived mixed-phase regime which at low *w* (<0.1 m s^−1^) corresponds to additional time on the order of several hours.

In these simulations, the first ice (Figure 2a) is observed at temperatures that correspond to small nucleation rate coefficients of approximately ∼1 cm^−3^ s^−1^ (see Figure 1). Hence, the simulated clouds are sensitive to *J* values that are more than 4 orders of magnitude below the range at which the majority of laboratory measurements have been made (*J* ≥ 10^4^ cm^−3^ s^−1^). This paucity of measurements in this regime of *J* ≤ 10^4^ cm^−3^ s^−1^ highlights a considerable gap in our current knowledge and the need for future laboratory studies to focus on homogeneous ice nucleation at higher temperatures.

Figure 2 also shows that the scatter in laboratory measurements, as represented by subsequent model parameterizations, has implications for the evolution of simulated mixed-phase clouds and their microphysical properties. In these simulations, all *J*_CNT_ parameterizations fall within the range of laboratory measurements of *J* and can be seen as conceivable representations; therefore, based on current measurements, it is not possible to determine which, if any, parameterization is more appropriate. Additional laboratory measurements at high and low temperatures would be required in order to establish the *T* dependence of *J*, a parameter which our study indicates is very important; such measurements would also help to define the absolute value of *J*.

## 4. Summary and Conclusions

In this study we used a parcel model with detailed cloud microphysics to show that the threshold freezing approximation, used in many microphysics schemes, is unable to suitably represent homogeneous freezing in liquid clouds. The evolution of cloud is sensitive to the finite rate of homogeneous nucleation well above −40°C, i.e., at temperatures where we traditionally assume only heterogeneous nucleation can produce ice in clouds. In some simulations, homogeneous freezing was active as warm as −30°C, which is considerably warmer (>8°C) than generally assumed to occur in clouds. A series of parameterizations based on CNT and constrained by the scatter in laboratory measurements was used to show that simulated clouds are sensitive to the chosen parameterization and therefore to the uncertainty in laboratory measurements. In particular, we found that the temperature dependence of the homogeneous ice nucleation rate coefficient is a key parameter for correctly determining the impact of homogeneous freezing on cloud properties. We recommend that future laboratory studies focus on nucleation at high (>−35°C) and low (<−38°C) temperatures in order to constrain a new parameterization.

The idealized simulations provide evidence for the unsuitability of using a threshold freezing temperature; however, it would be beneficial to extend this work to a three-dimensional spectral bin model where cloud scale interactions and feedbacks are included and explicitly represented. Moreover, inclusion of heterogeneous ice nucleation would also be required for understanding the competition between the two modes. Nevertheless, we recommend explicitly representing the temperature dependence of homogeneous ice nucleation rather than using a threshold approximation.

## References

[b1] Benz S, Megahed K, Möhler O, Saathoff H, Wagner R, Schurath U (2005). *T*-dependent rate measurements of homogeneous ice nucleation in cloud droplets using a large atmospheric simulation chamber. J. Photochem. Photobiol., A.

[b2] Boucher O (2013). Clouds and aerosols. In: Climate change 2013: The physical science basis. Contribution of Working Group I to the Fifth Assessment Report of the Intergovernmental Panel on Climate Change.

[b3] Butorin GT, Skripov VP (1972). Crystallization in supercooled water. Kristallografiya+.

[b4] Choi Y-S, Lindzen RS, Ho C-H, Kim J (2010). Space observations of cold-cloud phase change. Proc. Natl. Acad. Sci. U.S.A.

[b5] Cotton RJ, Field PR (2002). Ice nucleation characteristics of an isolated wave cloud. Q. J. R. Meteorol. Soc.

[b6] de Boer G, Morrison H, Shupe MD, Hildner R (2011). Evidence of liquid-dependent ice nucleation in high-latitude stratiform clouds from surface remote sensors. Geophys. Res. Lett.

[b7] Demott PJ (1990). An exploratory study of ice nucleation by soot aerosols. J. Appl. Meteorol.

[b8] DeMott PJ, Prenni AJ, Liu X, Kreidenweis SM, Petters MD, Twohy CH, Richardson MS, Eidhammer T, Rogers DC (2010). Predicting global atmospheric ice nuclei distributions and their impacts on climate. Proc. Natl. Acad. Sci. U.S.A.

[b9] Draginis M (1958). Liquid water within convective clouds. J. Meteorol.

[b10] Duft D, Leisner T (2004). Laboratory evidence for volume-dominated nucleation of ice in supercooled water microdroplets. Atmos. Chem. Phys.

[b11] Earle ME, Kuhn T, Khalizov AF, Sloan JJ (2010). Volume nucleation rates for homogeneous freezing in supercooled water microdroplets: Results from a combined experimental and modelling approach. Atmos. Chem. Phys.

[b12] Fan J, Comstock JM, Ovchinnikov M, McFarlane SA, McFarquhar G, Allen G (2010). Tropical anvil characteristics and water vapor of the tropical tropopause layer: Impact of heterogeneous and homogeneous freezing parameterizations. J. Geophys. Res.

[b13] Forbes RM, Ahlgrimm M (2014). On the representation of high-latitude boundary layer mixed-phase cloud in the ECMWF global model. Mon. Weather Rev.

[b14] Herbert RJ, Murray BJ, Whale TF, Dobbie SJ, Atkinson JD (2014). Representing time-dependent freezing behaviour in immersion mode ice nucleation. Atmos. Chem. Phys.

[b15] Hoyle CR (2011). Ice nucleation properties of volcanic ash from Eyjafjallajökull. Atmos. Chem. Phys.

[b16] Knopf DA, Rigg YJ (2011). Homogeneous ice nucleation from aqueous inorganic/organic particles representative of biomass burning: Water activity, freezing temperatures, nucleation rates. J. Phys. Chem. A.

[b17] Kong FY, Yau MK (1997). An explicit approach to microphysics in MC2. Atmos. Ocean.

[b18] Krämer B, Hübner O, Vortisch H, Wöste L, Leisner T, Schwell M, Rühl E, Baumgärtel H (1999). Homogeneous nucleation rates of supercooled water measured in single levitated microdroplets. J. Chem. Phys.

[b19] Ladino L, Stetzer O, Lüönd F, Welti A, Lohmann U (2011). Contact freezing experiments of kaolinite particles with cloud droplets. J. Geophy. Res.

[b20] Lim K-SS, Hong S-Y (2010). Development of an effective double-moment cloud microphysics scheme with prognostic cloud condensation nuclei (CCN) for weather and climate models. Mon. Weather Rev.

[b21] Lüönd F, Stetzer O, Welti A, Lohmann U (2010). Experimental study on the ice nucleation ability of size-selected kaolinite particles in the immersion mode. J. Geophys. Res.

[b22] Lynn BH, Khain AP, Dudhia J, Rosenfeld D, Pokrovsky A, Seifert A (2005). Spectral (bin) microphysics coupled with a mesoscale model (MM5): Part I. Model description and first results. Mon. Weather Rev.

[b23] Milbrandt JA, Yau MK (2005). A multimoment bulk microphysics parameterization: Part I. Analysis of the role of the spectral shape parameter. J. Atmos. Sci.

[b24] Morrison H, Curry JA, Khvorostyanov VI (2005). A new double-moment microphysics parameterization for application in cloud and climate models: Part I. Description. J. Atmos. Sci.

[b25] Murray BJ, Broadley SL, Wilson TW, Bull SJ, Wills RH, Christenson HK, Murray EJ (2010). Kinetics of the homogeneous freezing of water. Phys. Chem. Chem. Phys.

[b26] Murray BJ, O'Sullivan D, Atkinson JD, Webb ME (2012). Ice nucleation by particles immersed in supercooled cloud droplets. Chem. Soc. Rev.

[b27] Pruppacher HR (1995). A new look at homogeneous ice nucleation in supercooled water drops. J. Atmos. Sci.

[b28] Reisner J, Rasmussen RM, Bruintjes RT (1998). Explicit forecasting of supercooled liquid water in winter storms using the MM5 mesoscale model. Q. J. R. Meteorol. Soc.

[b29] Riechers B, Wittbracht F, Hutten A, Koop T (2013). The homogeneous ice nucleation rate of water droplets produced in a microfluidic device and the role of temperature uncertainty. Phys. Chem. Chem. Phys.

[b30] Rosenfeld D, Lensky IM (1998). Satellite-based insights into precipitation formation processes in continental and maritime convective clouds. Bull. Am. Meteorol. Soc.

[b31] Rosenfeld D, Woodley WL (2000). Deep convective clouds with sustained supercooled liquid water down to −37.5 degrees C. Nature.

[b32] Rzesanke D, Nadolny J, Duft D, Muller R, Kiselev A, Leisner T (2012). On the role of surface charges for homogeneous freezing of supercooled water microdroplets. Phys. Chem. Chem. Phys.

[b33] Seifert A, Beheng KD (2006). A two-moment cloud microphysics parameterization for mixed-phase clouds: Part 1. Model description. Meteorol. Atmos. Phys.

[b34] Shipway BJ, Hill AA (2012). Diagnosis of systematic differences between multiple parametrizations of warm rain microphysics using a kinematic framework. Q. J. R. Meteorol. Soc.

[b35] Stan CA, Schneider GF, Shevkoplyas SS, Hashimoto M, Ibanescu M, Wiley BJ, Whitesides GM (2009). A microfluidic apparatus for the study of ice nucleation in supercooled water drops. Lab Chip.

[b36] Stöckel P, Weidinger IM, Baumgartel H, Leisner T (2005). Rates of homogeneous ice nucleation in levitated H_2_O and D_2_O droplets. J. Phys. Chem. A.

[b37] Taborek P (1985). Nucleation in emulsified supercooled water. Phys. Rev. B: Condens. Matter.

[b38] Thompson G, Field PR, Rasmussen RM, Hall WD (2008). Explicit forecasts of winter precipitation using an improved bulk microphysics scheme. Part II: Implementation of a new snow parameterization. Mon. Weather Rev.

[b39] Vali G (2014). Interpretation of freezing nucleation experiments: Singular and stochastic sites and surfaces. Atmos. Chem. Phys.

[b40] Walko RL, Cotton WR, Meyers MP, Harrington JY (1995). New RAMS cloud microphysics parameterization: Part I. The single-moment scheme. Atmos. Res.

[b41] Westbrook CD, Illingworth AJ (2011). Evidence that ice forms primarily in supercooled liquid clouds at temperatures >−27°C. Geophys. Res. Lett.

[b42] Wood GR, Walton AG (1970). Homogeneous nucleation kinetics of ice from water. J. Appl. Phys.

[b43] Wood SE, Baker MB, Swanson BD (2002). Instrument for studies of homogeneous and heterogeneous ice nucleation in free-falling supercooled water droplets. Rev. Sci. Instrum.

[b44] Wright TP, Petters MD (2013). The role of time in heterogeneous freezing nucleation. J. Geophys. Res. Atmos.

[b45] Zobrist B, Koop T, Luo BP, Marcolli C, Peter T (2007). Heterogeneous ice nucleation rate coefficient of water droplets coated by a nonadecanol monolayer. J. Phys. Chem. C.

