# Supplementary material for: Sensitivity of liquid clouds to homogenous freezing parameterizations
Source: Geophys Res Lett. 2015 Mar 13;42(5):1599–605. doi: 10.1002/2014GL062729 (PMC4459198; doi:10.1002/2014GL062729)
Supplement: Supplementary file 1 — Tables S1 and S2 and Figure S1 [file grl0042-1599-sd1.docx]

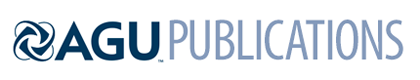


*Geophysical Research Letters*

Supporting Information for

**Sensitivity of liquid clouds to homogenous freezing parameterisations**

R.J. Herbert^1^, B.J.Murray^1^, T. Koop^2^, S.J. Dobbie^1^

^1^School of Earth and Environment, University of Leeds, Leeds, UK, ^2^Faculty of Chemistry, Bielefeld University, Bielefeld, Germany

**Contents of this file**

Figure S1

Tables S1 to S2

**Introduction**

The supporting information contains the following:

- Figure S1 is for an identical set of simulations as that shown in Figure 1 of the manuscript, except that the simulation was initiated at -30 °C rather than -5 °C. For clarity, the scale of the cloud ice effective radius (panel b) has been adjusted, and also the scale of the total cloud mass (panel e).
- Table S1 is a review of microphysics schemes that simulate both liquid and ice phase from the literature. Only studies that describe a new microphysics scheme or a major addition to an existing microphysics scheme are included. From each study we determined the homogeneous parameterisation employed in each scheme. In the event that this was not detailed we have marked the study as “No homogenous freezing”. We also list the model that the microphysics scheme is associated with and also the scale that the model is used for including parcel model, cloud resolving model (CRM), mesoscale model, Numerical Weather Prediction model (NWP) or Global Climate Model (GCM).
- Table S2 shows the equations used for the four temperature-dependent parameterisations shown in Figure 1 of the manuscript.

Figure S1. Same as Figure 2 but for simulations initiated at -30 °C. Simulated one dimensional evolution of cloud variables as a function of constant updraught speed using different homogeneous freezing representations. Variables include: (a) cloud ice particle number concentration; (b) cloud ice particle effective radius (expression for the cross-section area weighted mean radius); (c) snow mass mixing ratio; (d) cloud ice fraction (ratio of ice mass mixing ratio to total hydrometeor mass mixing ratio); and (e) total cloud mass mixing ratio (all ice and water species). The first four columns demonstrate sensitivity of the cloud to the laboratory-constrained *J*(*T*) parameterisations, and the final column shows simulations using a threshold approximation of -40 °C at which point all liquid instantly freezes.

| Model | Scale | Microphysics scheme | Homogeneous parameterisation used |
| --- | --- | --- | --- |
|  |  |  |  |
| CAM-OSLO | GCM | *Storelvmo et al.* [2008] | Threshold at -40 °C |
| CAM5 |  | *Neale et al.* [2010] | Thresholds at -40 °C (cloud droplets) & -5 °C (rain) |
| CAM3.5 |  | *Song and Zhang* [2011] | Threshold at -40 °C |
|  |  |  |  |
| COSMO | Mesoscale | *Doms et al.* [2005] | Threshold at -37 °C |
|  |  |  |  |
| CSU RAMS | CRM | *Walko et al.* [1995] | *T*-dependent CNT-based [*DeMott et al.*, 1994] |
|  |  |  |  |
| ECHAM6 | GCM | *Lohmann and Roeckner* [1996] | Threshold at -35 °C |
|  |  |  |  |
| ECMWF | GCM | *Forbes and Ahlgrimm* [2014] | Threshold at -38 °C |
|  |  |  |  |
| GCE | CRM/Mesoscale | *Tao et al.* [2003] | *T*-dependent CNT-based [*Pruppacher*, 1995] |
| '' | Mesoscale | *Tao and Simpson* [1993] | Threshold at -40 °C |
|  |  |  |  |
| GEOS-5 | GCM | *Barahona et al.* [2014] | Threshold at -38 °C |
|  |  |  |  |
| GSR | CRM | *Straka and Mansell* [2005] | Threshold at -40 °C |
|  |  |  |  |
| HUCM | CRM | *Khain et al.* [2004] | *T*-dependent CNT-based [*Pruppacher*, 1995] |
|  |  |  |  |
| MAC3 | CRM | *Yin et al.* [2000] | No homogeneous freezing |
|  |  |  |  |
| MC2 | Mesoscale | *Kong and Yau* [1997] | Threshold at -40 °C |
|  |  |  |  |
| MetOffice LEM | CRM | *Gray et al.* [2001] | Threshold at -38 °C |
|  |  |  |  |
| MM4 | Mesoscale | *Mölders et al.* [1994] | Threshold at -35 °C |
| MM5 |  | *Reisner et al.* [1998] | Threshold at -40 °C |
| MM5 |  | *Grell et al.* [1994] | Threshold at -40 °C |
| MM5 (SBM) |  | *Lynn et al.* [2005] | *T*-dependent CNT-based [*Pruppacher*, 1995] |
|  |  |  |  |
| Straka Atm. Model | CRM | *Gilmore et al.* [2004] | Threshold at -40 °C for cloud water only |
|  |  |  |  |
| Sys. Atm. Model | CRM | *Fan et al.* [2009] | *T*-dependent below -36 °C [*Bigg,* 1953] |
|  |  |  |  |
| SHIPS / UWNMS | Mesoscale | *Hashino and Tripoli* [2008] | *T*-dependent [*Heymsfield and Miloshevich*, 1993] |
|  |  |  |  |
| WRF | CRM/Mesoscale/GCM | WSM3/5 *Hong et al.* [2004] | Threshold at -40 °C |
| '' |  | *Milbrandt and Yau* [2005] | *T*-dependent CNT-based [*DeMott et al.*, 1994] |
| '' |  | *Morrison et al.* [2005] | Threshold at -40 °C |
| '' |  | WSM6 *Hong and Lim* [2006] | Threshold at -40 °C |
| '' |  | *Phillips et al.* [2007] | Thresholds at ~ -36 °C (cloud droplets) & -35 °C (rain) |
| '' |  | *Thompson et al.* [2008] | Threshold at -38 °C |
| '' |  | WDM6 *Lim and Hong* [2010] | Threshold at -40 °C |
| '' |  | *Thompson and Eidhammer* [2014] | Threshold at -38 °C |
|  |  |  |  |
| None | CRM/Mesoscale | *Rutledge and Hobbs* [1983] | No homogeneous freezing |
| '' | CRM | *Lin et al.* [1983] | Threshold at -40 °C |
| '' | CRM | *Lord et al.* [1984] | Threshold at -40 °C |
| '' | CRM | *Ziegler* [1985] | Threshold at -40 °C |
| '' | CRM | *Murakami* [1990] | Threshold at -40 °C |
| '' | CRM | *Wang and Chang* [1993] | Threshold at -40 °C |
| '' | Parcel Model | *Cotton and Field* [2002] | *T*-dependent CNT-based [*Jeffery and Austin*, 1997] |
| '' | CRM/Mesoscale | *Seifert and Beheng* [2006] | *T*-dependent CNT-based [*Jeffery and Austin*, 1997] |
| '' | Parcel Model | *Eidhammer et al.* [2009] | *T*-dependent and RH dependent [*Koop*, 2000] |
| '' | Parcel Model | *Ervens and Feingold* [2012] | "Homogeneous not considered" |

**Table S1**. A review of microphysics schemes which include both liquid and ice phases. The majority of schemes describe homogeneous freezing of pure liquid droplets using a threshold freezing temperature of -40 °C.

|  | Equation |
| --- | --- |
| *J*_Prup_ | $\begin{matrix} \log_{10}\left(J_{\text{Prup}}\right)=176.871+8366.461T-140.0691784T^{2} \\ +0.8789001T^{3}-2.449853\times10^{-3}T^{4}+2.5594176437\times10^{-6}T^{5} \end{matrix}$ |
| *J*_Prup-Low_ | $\begin{matrix} \log_{10}\left(J_{\text{Prup-Low}}\right)=175.0886+8238.3122T-138.6505958T^{2} \\ +0.874579557T^{3}-2.45063431\times10^{-3}T^{4}+2.5736888\times10^{-6}T^{5} \end{matrix}$ |
| *J*_Zob_ | $\log_{10}\left(J_{\text{Zob}}\right)=45705.562-601.7263T+2.6465459T^{2}-3.886976\times10^{-3}T^{3}$ |
| *J*_Zob-Shallow_ | $\log_{10}\left(J_{\text{Zob-Shallow}}\right)=520.871-15.2227T+0.1053487T^{2}-2.12124\times10^{-4}T^{3}$ |

**Table S2**. Parameterisations used for the homogeneous nucleation rate coefficient (*J*) shown in Figure 1

References

# Barahona, D., A. Molod, J. Bacmeister, A. Nenes, A. Gettelman, H. Morrison, V. Phillips, and A. Eichmann (2014), Development of two-moment cloud microphysics for liquid and ice within the NASA Goddard Earth Observing System Model (GEOS-5), *Geosci. Model Dev.*, *7*(4), 1733-1766, doi: 10.5194/gmd-7-1733-2014.

# Bigg, E. K. (1953), The formation of atmospheric ice crystals by the freezing of droplets, *Q. J. R. Meteorol. Soc.*, *79*(342), 510-519, doi: 10.1002/qj.49707934207.

Cotton, R. J., and P. R. Field (2002), Ice nucleation characteristics of an isolated wave cloud, *Q. J. R. Meteorol. Soc.*, *128*(585), 2417-2437, doi: 10.1256/qj.01.150.

DeMott, P. J., M. P. Meyers, and W. R. Cotton (1994), Parameterization and Impact of Ice initiation Processes Relevant to Numerical Model Simulations of Cirrus Clouds, *J. Atmos. Sci.*, *51*(1), 77-90, doi: doi:10.1175/1520-0469(1994)051<0077:PAIOII>2.0.CO;2.

Eidhammer, T., P. J. DeMott, and S. M. Kreidenweis (2009), A comparison of heterogeneous ice nucleation parameterizations using a parcel model framework, *J. Geophys. Res.*, *114*(D6), D06202, doi: 10.1029/2008jd011095.

Ervens, B., and G. Feingold (2012), On the representation of immersion and condensation freezing in cloud models using different nucleation schemes, *Atmos. Chem. Phys.*, *12*(13), 5807-5826, doi: 10.5194/acp-12-5807-2012.

# Fan, J., M. Ovtchinnikov, J. M. Comstock, S. A. McFarlane, and A. Khain (2009), Ice formation in Arctic mixed-phase clouds: Insights from a 3-D cloud-resolving model with size-resolved aerosol and cloud microphysics, *Journal of Geophysical Research: Atmospheres*, *114*(D4), D04205, doi: 10.1029/2008JD010782.

Forbes, R. M., and M. Ahlgrimm (2014), On the Representation of High-Latitude Boundary Layer Mixed-Phase Cloud in the ECMWF Global Model, *Monthly Weather Review*, *142*(9), 3425-3445, doi: 10.1175/MWR-D-13-00325.1.

Gilmore, M. S., J. M. Straka, and E. N. Rasmussen (2004), Precipitation and Evolution Sensitivity in Simulated Deep Convective Storms: Comparisons between Liquid-Only and Simple Ice and Liquid Phase Microphysics, *Monthly Weather Review*, *132*(8), 1897-1916, doi: 10.1175/1520-0493(2004)132<1897:PAESIS>2.0.CO;2.

Gray, M. E. B., J. Petch, S. H. Derbyshire, A. R. Brown, A. P. Lock, H. A. Swann, and P. R. A. Brown (2001), Version 2.3 of theMet. Office large eddy model: Part II Scientific documentation, The MetOffice, FitzRoy Road, Exeter EX1 3PB, UK.

Grell, G. A., J. Dudhia, and D. Stauffer (1994), A Description of the Fifth-Generation Penn State/NCAR Mesoscale Model (MM5), Boulder, Colo.

Hashino, T., and G. J. Tripoli (2008), The Spectral Ice Habit Prediction System (SHIPS). Part II: Simulation of Nucleation and Depositional Growth of Polycrystals, *J. Atmos. Sci.*, *65*(10), 3071-3094, doi: 10.1175/2008JAS2615.1.

Heymsfield, A. J., and L. M. Miloshevich (1993), Homogeneous Ice Nucleation and Supercooled Liquid Water in Orographic Wave Clouds, *J. Atmos. Sci.*, *50*(15), 2335-2353, doi: doi:10.1175/1520-0469(1993)050<2335:HINASL>2.0.CO;2.

Hong, S.-Y., and J.-O. Lim (2006), The WRF Single-Moment 6-Class Microphysics Scheme (WSM6), *J. Korean Meteor. Soc.*, *42*(2), 129-151.

Hong, S.-Y., J. Dudhia, and S.-H. Chen (2004), A Revised Approach to Ice Microphysical Processes for the Bulk Parameterization of Clouds and Precipitation, *Monthly Weather Review*, *132*(1), 103-120, doi: 10.1175/1520-0493(2004)132<0103:ARATIM>2.0.CO;2.

Jeffery, C. A., and P. H. Austin (1997), Homogeneous nucleation of supercooled water: Results from a new equation of state, *Journal of Geophysical Research: Atmospheres*, *102*(D21), 25269-25279, doi: 10.1029/97JD02243.

Khain, A., A. Pokrovsky, M. Pinsky, A. Seifert, and V. Phillips (2004), Simulation of Effects of Atmospheric Aerosols on Deep Turbulent Convective Clouds Using a Spectral Microphysics Mixed-Phase Cumulus Cloud Model. Part I: Model Description and Possible Applications, *J. Atmos. Sci.*, *61*(24), 2963-2982, doi: 10.1175/JAS-3350.1.

Kong, F. Y., and M. K. Yau (1997), An explicit approach to microphysics in MC2, *Atmosphere-Ocean*, *35*(3), 257-291, doi: 10.1080/07055900.1997.9649594.

Koop, T. (2000), The formation of ice clouds from supercooled aqueous aerosols, *Nucleation and Atmospheric Aerosols 2000*, *534*, 549-560.

Lim, K.-S. S., and S.-Y. Hong (2010), Development of an Effective Double-Moment Cloud Microphysics Scheme with Prognostic Cloud Condensation Nuclei (CCN) for Weather and Climate Models, *Monthly Weather Review*, *138*(5), 1587-1612, doi: 10.1175/2009mwr2968.1.

Lin, Y.-L., R. D. Farley, and H. D. Orville (1983), Bulk Parameterization of the Snow Field in a Cloud Model, *Journal of Climate and Applied Meteorology*, *22*(6), 1065-1092, doi: 10.1175/1520-0450(1983)022<1065:BPOTSF>2.0.CO;2.

# Lohmann, U., and E. Roeckner (1996), Design and performance of a new cloud microphysics scheme developed for the ECHAM general circulation model, *Climate Dynamics*, *12*(8), 557-572, doi: 10.1007/BF00207939.

Lord, S. J., H. E. Willoughby, and J. M. Piotrowicz (1984), Role of a Parameterized Ice-Phase Microphysics in an Axisymmetric, Nonhydrostatic Tropical Cyclone Model, *J. Atmos. Sci.*, *41*(19), 2836-2848, doi: 10.1175/1520-0469(1984)041<2836:ROAPIP>2.0.CO;2.

Lynn, B. H., A. P. Khain, J. Dudhia, D. Rosenfeld, A. Pokrovsky, and A. Seifert (2005), Spectral (Bin) Microphysics Coupled with a Mesoscale Model (MM5). Part I: Model Description and First Results, *Monthly Weather Review*, *133*(1), 44-58, doi: 10.1175/MWR-2840.1.

Milbrandt, J. A., and M. K. Yau (2005), A Multimoment Bulk Microphysics Parameterization. Part I: Analysis of the Role of the Spectral Shape Parameter, *J. Atmos. Sci.*, *62*(9), 3051-3064, doi: 10.1175/JAS3534.1.

Mölders, N., H. Hass, H. J. Jakobs, M. Laube, and A. Ebel (1994), Some Effects of Different Cloud Parameterizations in a Mesoscale Model and a Chemistry Transport Model, *J. App. Meteorol.*, *33*(4), 527-545, doi: 10.1175/1520-0450(1994)033<0527:SEODCP>2.0.CO;2.

Morrison, H., J. A. Curry, and V. I. Khvorostyanov (2005), A new double-moment microphysics parameterization for application in cloud and climate models. Part I: Description, *J. Atmos. Sci.*, *62*(6), 1665-1677, doi: 10.1175/Jas3446.1.

Murakami, M. (1990), Numerical Modeling of Dynamical and Microphysical Evolution of an Isolated Convective Cloud - The 19 July 1981 CCOPE Cloud -, *Journal of the Meteorological Society of Japan. Ser. II*, *68*(2), 107-128.

Neale, R. B., et al. (2010), Description of the NCAR Community Atmosphere Model (CAM 5.0), NCAR, Boulder, Colo.

# Phillips, V. T. J., L. J. Donner, and S. T. Garner (2007), Nucleation processes in deep convection simulated by a cloud-system-resolving model with double-moment bulk microphysics, *J. Atmos. Sci.*, *64*(3), 738-761, doi: Doi 10.1175/Jas3869.1.

Pruppacher, H. R. (1995), A New Look at Homogeneous Ice Nucleation in Supercooled Water Drops, *J. Atmos. Sci.*, *52*(11), 1924-1933, doi: 10.1175/1520-0469(1995)052<1924:Anlahi>2.0.Co;2.

Reisner, J., R. M. Rasmussen, and R. T. Bruintjes (1998), Explicit forecasting of supercooled liquid water in winter storms using the MM5 mesoscale model, *Q. J. R. Meteorol. Soc.*, *124*(548), 1071-1107, doi: 10.1002/qj.49712454804.

Rutledge, S. A., and P. Hobbs (1983), The Mesoscale and Microscale Structure and Organization of Clouds and Precipitation in Midlatitude Cyclones. VIII: A Model for the “Seeder-Feeder” Process in Warm-Frontal Rainbands, *J. Atmos. Sci.*, *40*(5), 1185-1206, doi: 10.1175/1520-0469(1983)040<1185:TMAMSA>2.0.CO;2.

Seifert, A., and K. D. Beheng (2006), A two-moment cloud microphysics parameterization for mixed-phase clouds. Part 1: Model description, *Meteorol. Atmos. Phys.*, *92*(1-2), 45-66, doi: 10.1007/s00703-005-0112-4.

Song, X., and G. J. Zhang (2011), Microphysics parameterization for convective clouds in a global climate model: Description and single-column model tests, *Journal of Geophysical Research: Atmospheres*, *116*(D2), D02201, doi: 10.1029/2010JD014833.

Storelvmo, T., J. E. Kristjánsson, and U. Lohmann (2008), Aerosol Influence on Mixed-Phase Clouds in CAM-Oslo, *J. Atmos. Sci.*, *65*(10), 3214-3230, doi: 10.1175/2008JAS2430.1.

Straka, J. M., and E. R. Mansell (2005), A Bulk Microphysics Parameterization with Multiple Ice Precipitation Categories, *J. App. Meteorol.*, *44*(4), 445-466, doi: 10.1175/JAM2211.1.

Tao, W. K., and J. Simpson (1993), The Goddard Cumulus Ensemble Model. Part I: Model description, *Terrestrial, Atmospheric and Oceanic Sciences*, *4*, 19-54.

Tao, W. K., et al. (2003), Microphysics, radiation and surface processes in the Goddard Cumulus Ensemble (GCE) model, *Meterol. Atmos. Phys.*, *82*, 97-137, doi: 10.1007/s00703-001-0594-7.

Thompson, G., and T. Eidhammer (2014), A Study of Aerosol Impacts on Clouds and Precipitation Development in a Large Winter Cyclone, *J. Atmos. Sci.*, *71*(10), 3636-3658, doi: 10.1175/JAS-D-13-0305.1.

Thompson, G., P. R. Field, R. M. Rasmussen, and W. D. Hall (2008), Explicit Forecasts of Winter Precipitation Using an Improved Bulk Microphysics Scheme. Part II: Implementation of a New Snow Parameterization, *Monthly Weather Review*, *136*(12), 5095-5115, doi: 10.1175/2008mwr2387.1.

Walko, R. L., W. R. Cotton, M. P. Meyers, and J. Y. Harrington (1995), New RAMS cloud microphysics parameterization part I: the single-moment scheme, *Atmos. Res.*, *38*(1–4), 29-62, doi: http://dx.doi.org/10.1016/0169-8095(94)00087-T.

Wang, C., and J. S. Chang (1993), A three-dimensional numerical model of cloud dynamics, microphysics, and chemistry: 1. Concepts and formulation, *Journal of Geophysical Research: Atmospheres*, *98*(D8), 14827-14844, doi: 10.1029/92JD01393.

Yin, Y., Z. Levin, T. G. Reisin, and S. Tzivion (2000), The effects of giant cloud condensation nuclei on the development of precipitation in convective clouds — a numerical study, *Atmos. Res.*, *53*(1–3), 91-116, doi: http://dx.doi.org/10.1016/S0169-8095(99)00046-0.

Ziegler, C. L. (1985), Retrieval of Thermal and Microphysical Variables in Observed Convective Storms. Part 1: Model Development and Preliminary Testing, *J. Atmos. Sci.*, *42*(14), 1487-1509, doi: 10.1175/1520-0469(1985)042<1487:ROTAMV>2.0.CO;2.
